# Supplementary material for: ChimeraTE: a pipeline to detect chimeric transcripts derived from genes and transposable elements
Source: Nucleic Acids Res. 2023 Aug 24;51(18):9764–84. doi: 10.1093/nar/gkad671 (PMC10570057; doi:10.1093/nar/gkad671)
Supplement: gkad671_Supplemental_Files [file gkad671_supplemental_files.zip › supData_OliveiraDS-03.docx]

**Suplementary Data**

**Sup. Note 1:** Differences between Mode 1 and Mode 2………………………………………………………………1

**Sup. Note 2:** Replicability and coverage of chimeric transcripts in both ChimeraTE modes………..5

**Sup. Note 3:** Artifactual chimeric reads are largely reduced with RNA-seq replicability……………...7

**Note 1: Differences between Mode 1 and Mode 2**

ChimeraTE Mode 1 and Mode 2 use different alignment strategies and downstream approaches to detect chimeric transcripts (see Methods). In order to test whether these differences can lead to distinct outputs, we compared chimeras from Mode 1 and Mode 2 by using the same RNA-seq libraries from four *D. melanogaster* wild-type strains. Taking all strains together, Mode 2 uncovered 171 chimeric transcripts generated by 165 out of the 327 genes (50.46%) identified by Mode 1, representing 25% of all TE-initiated transcripts detected by Mode 1; ~52% of TE-exonized; ~29% of TE-terminated (Figure S1.1A). These results indicate that ChimeraTE Mode 2 had low sensitivity (27.39%) to detect chimeric transcripts from TE insertions near genes. For chimeras derived from TE inside genes, 17.23% of them were detected by the transcriptome assembly approach in Mode 2, showing the relevance of performing this optional analysis. However, it must be considered that *--assembly* performed by Mode 2 is time-consuming, as well as hardware-consuming (Table S1.1).


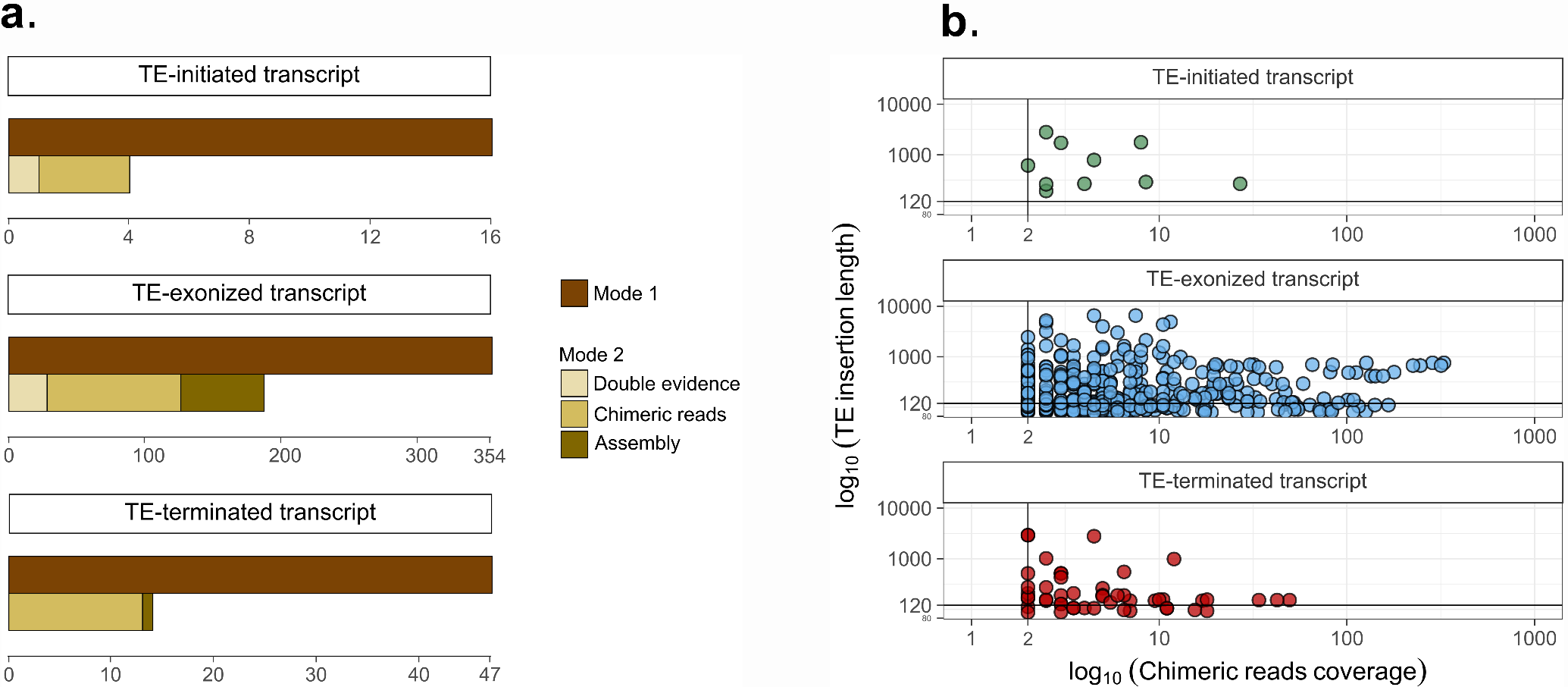


| **Species** | **Reads** | **Mode 1** | **Mode 2** |  |
| --- | --- | --- | --- | --- |
|  |  |  |  |  |
|  |  | ***Time*** | ***Time*** |  |
| dmgoth101 | 36M | 01h:50min | 11h:25min |  |
| dmgoth63 | 40M | 01h:55min | 14h:20min |  |
| dmsj23 | 35M | 01h:30min | 11h:35min |  |
| dmsj7 | 42M | 02h:00min | 13h:35min |  |
| *H. sapiens* | 58M | 1d 3h:00min | 3d13h:40min |  |
| *A. thaliana* | 45M | 02h:32min | 19h:10min |  |
| *Poecilia reticulata* | 49M | 3h:20min | 1d 10h:05min |  |

**Figure S1.1**: **A**) Total chimeric transcripts detected by ChimeraTE mode 1 and ChimeraTE Mode 2. The three brown boxes depict the type of evidence used by Mode 2 to support the chimeric transcripts (see Methods). Mode 2 had more efficiency to detect chimeric transcripts derived from TEs inside exons than near genes. **B**) Chimeric transcripts found by Mode 1, but not by Mode 2. 47.55% of all chimeric transcripts detected only by Mode 1 have TEs shorter than the read length (120 nt), and 32.51% of chimeras with TEs longer than reads have low chimeric reads coverage (10 chimeric reads). These factors explain the differences between results found by both Modes.

**Table S1.1**: Running time of ChimeraTE Mode 1 and Mode 2 (with --assembly option), in the four *D. melanogaster* wild-type strains, human, A. thaliana, and *P. reticulata*. All species were analyzed with two RNA-seq replicates. The analysis was performed with --threads 32 cores and 64Gb RAM (--ram 64 for Mode 2 --assembly).

In both ChimeraTE Modes, the main evidence used to detect chimeric transcripts is the presence of chimeric reads, which are paired-end reads spanning between TE and gene sequences. In Mode 1, at least 50% of one read (default parameter) from the read pair must align against the TE insertion, whereas in Mode 2 the whole read must align against a TE copy*.* Therefore, the alignment method performed by Mode 2 does not allow the detection of chimeric transcripts derived from TE copies shorter than the read size. Hence, we investigated whether chimeric transcripts found with Mode 1, but not with Mode 2, are generated by TE insertions shorter than the sequenced reads. From 237 chimeric transcripts predicted only by Mode 1, we found that 77 (~32%) have TEs shorter than the read size (120 nt), making it impossible to detect them with Mode 2 (Figure S1.1B). It is important to highlight that TEs longer than reads may have splice sites, generating chimeric transcripts with a small TE-derived fragment, not being detected by Mode 2 as well. Furthermore, we observed from Mode 2 results that 121 (~57%) chimeric transcripts from TEs longer than the reads have coverage lower than 10 chimeric reads. We hypothesized that Mode 1 may have substantially more TE- aligned reads than Mode 2, due to the use of strain-specific TE insertions and by counting split read alignment.

ChimeraTE Mode 1 is dependent on a reliable genome annotation for both genes and TEs, contrary to Mode 2. Indeed, we found in total, seven chimeric transcripts detected only with Mode 2 (Table S1.2), which are derived from genes that were not annotated in the wild-type genomes. We compared them with the *dm6* genome and we found all seven have the predicted TE family inserted near/inside the gene, reinforcing these annotations (Figure S1.2). For instance, the chimeric transcript *CG3164-McClintock* was detected by Mode 2 with double evidence in the four wild-type strains, and it is not annotated in any of the four genomes, perhaps due to its location in the telomeric region of the 2L chromosome (Figure S1.2G).

| **dmgoth101** | | **dmgoth63** | | **dmsj23** | | **dmsj7** | |
| --- | --- | --- | --- | --- | --- | --- | --- |
| **Gene** | **TE** | **Gene** | **TE** | **Gene** | **TE** | **Gene** | **TE** |
| FBgn0287182 | transib2 | FBgn0003016 | jockey | FBgn0286778 | HMS-Beagle | FBgn0042127 | roo |
| FBgn0287183 | roo | FBgn0287182 | transib2 | FBgn0287183 | rover | FBgn0287182 | transib2 |
| FBgn0288229 | McClintock | FBgn0288229 | McClintock | FBgn0288229 | McClintock | FBgn0288229 | McClintock |
| FBgn0286778 | roo | FBgn0286778 | HMS-Beagle | FBgn0287478 | roo |  |  |
|  |  | FBgn0287478 | roo |  |  |  |  |

**Table S1.2**: Chimeric transcripts found by Mode 2, but not by Mode 1, due to lack of genomic annotation.

**
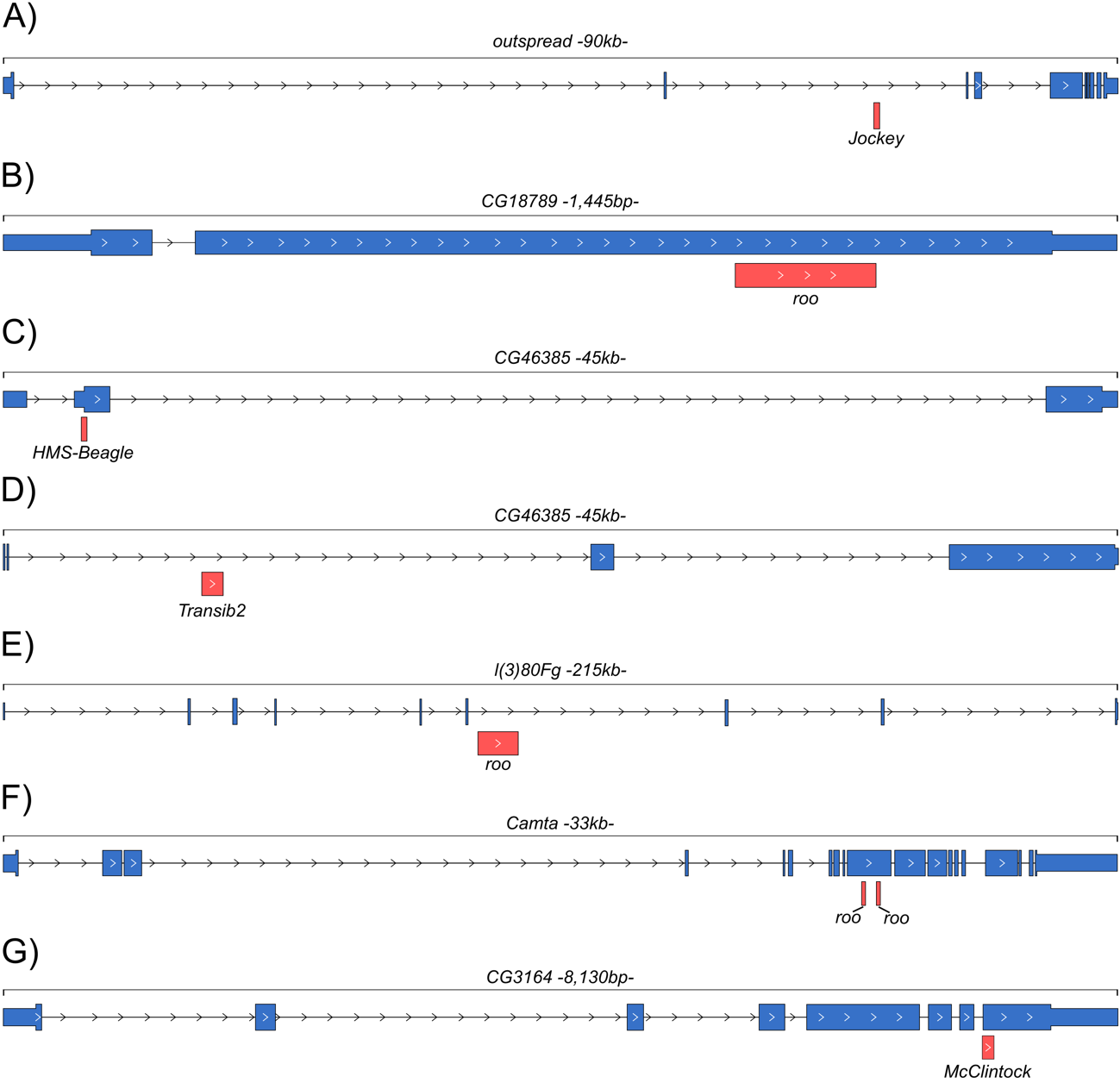
Figure S1.2**: Gene and TE annotation from *dm6* genome for chimeric transcripts identified by Mode 2 corresponding to genes that were not annotated in the wild-type genome assemblies. Blue boxes: UTRs and CDSs; Red boxes: TE insertion identified by ChimeraTE as the TE family generating the chimeric transcript.

ChimeraTE Mode 1 and Mode 2 detected 327 and 324 genes producing chimeric transcripts respectively, of which 165 were found by both methods. In Mode 2, chimeras with TE-derived sequences smaller than the read length are not detected. However, Mode 2 can detect TEs that are absent from the reference genome, along with low-frequency TE insertions of the wild-type strains at the population level, which are not present in the assembled Nanopore genome. Since low-frequency Nanopore reads are discarded during the genome assembly (54), Mode 1 is not able to detect them, whereas Mode 2 can. Furthermore, Mode 1 detects chimeric transcripts derived from TEs inside or +/- 3 kb from genes. TEs located farther away from genes and involved in chimeric transcripts are not detected. Such chimeras have been reported in *D. melanogaster* (31), as well as TEs acting as distal *cis*-regulatory elements (70, 71). Therefore, although we could consider them as potential false positives all cases in which the TE was not found inside/near a gene in the manual curation, we speculate that part of these cases found only by Mode 2 might be either from low-frequency TEs in the pool of individuals sequenced, or from TEs located far from genes.

**Note 2: Replicability and coverage of chimeric transcripts in both ChimeraTE modes**

The ability to detect TE expression is an important factor to identify chimeric reads. The different strategies of alignment to identify chimeric reads in Mode 1 and Mode 2 cause differences in the sensitivity of chimeric transcript detection (Figure 2.1A). We investigated whether such differences might be associated with the detection of TE-derived reads. We found that Mode 1 is more efficient than Mode 2 in detecting reads aligned against TE insertions (Figure S2.2), as well as for chimeric read detection (Figure 2.1A). Indeed, the proportion of chimeric reads in Mode 1 and Mode 2 is 0.34% and 0.04% of the total library sizes, respectively. The power of chimeric read detection from the two Modes is different because of the alignment strategies used. Despite the differences, both modes show significant positive correlations between the library size 45and the number of chimeric reads, as well as the number of TE-aligned reads and chimeric reads (Figure S2.3).


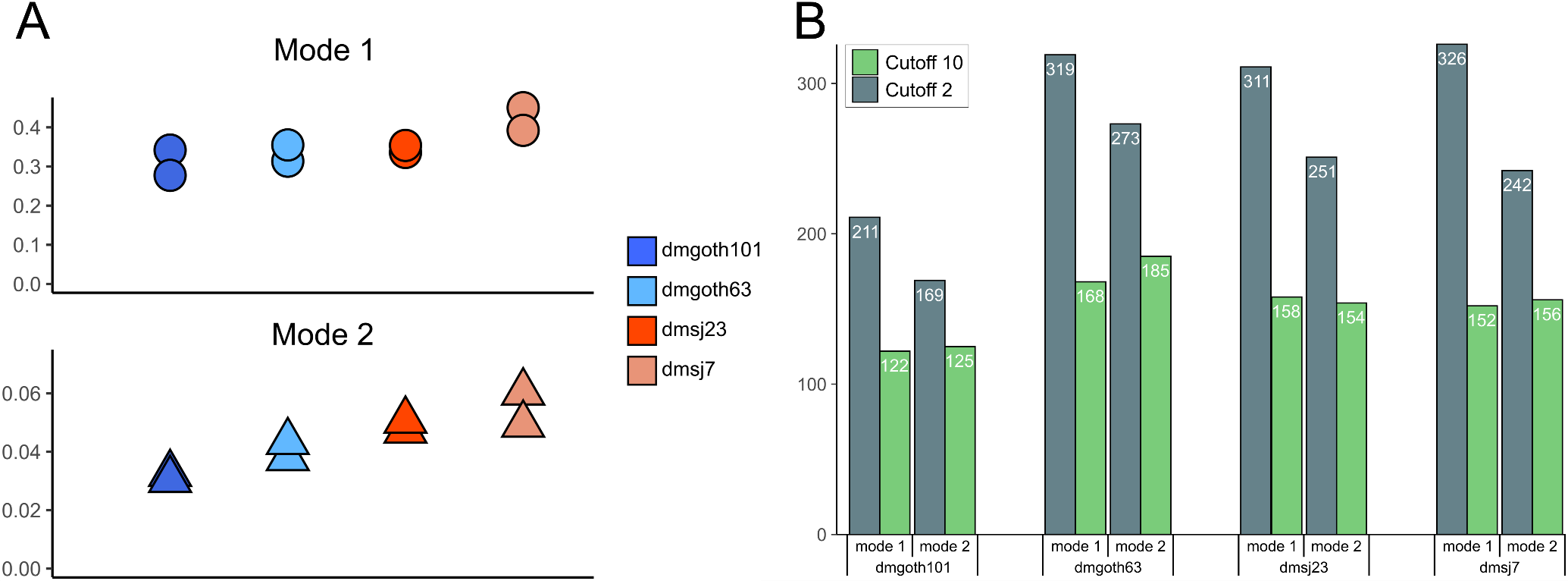


**Figure S2.1**: **A**) Proportion of reads from RNA-seq libraries comprised by chimeric reads on both replicates. **B**) Chimeric transcripts detected by both modes of ChimeraTE. The bars represent chimeras found in both RNA-seq replicates in the four *D. melanogaster* wild-type strains, applying two chimeric read cutoffs: 2 and 10.


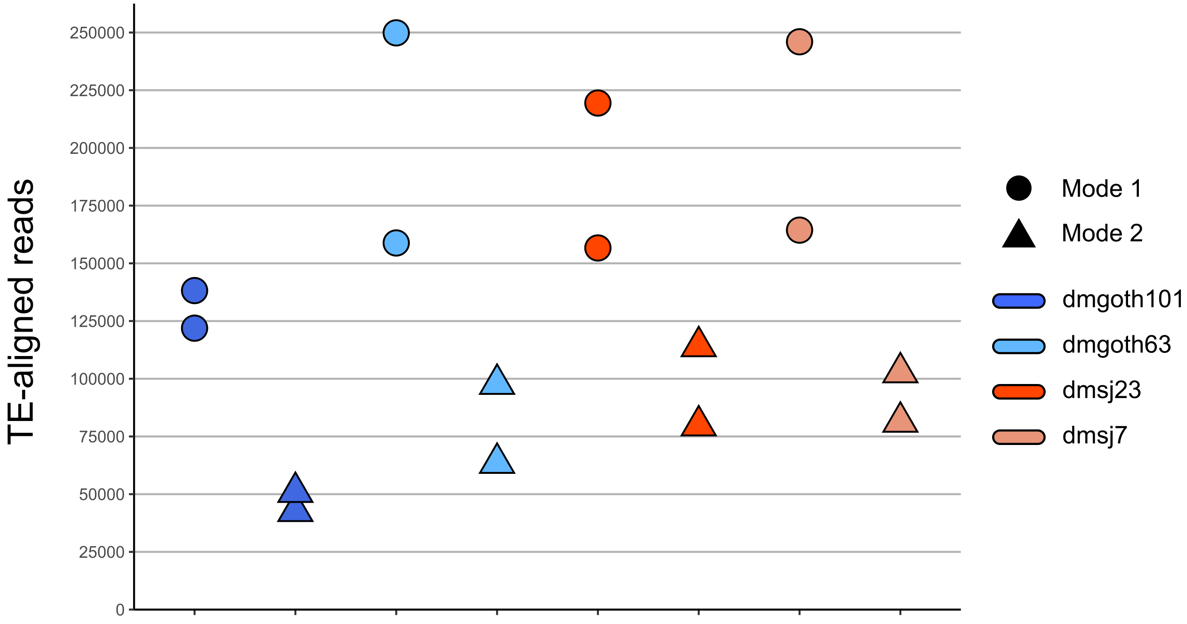


**Figure S2.2**: The total number of TE-aligned reads between both ChimeraTE Modes, in all strains and their respective replicates.


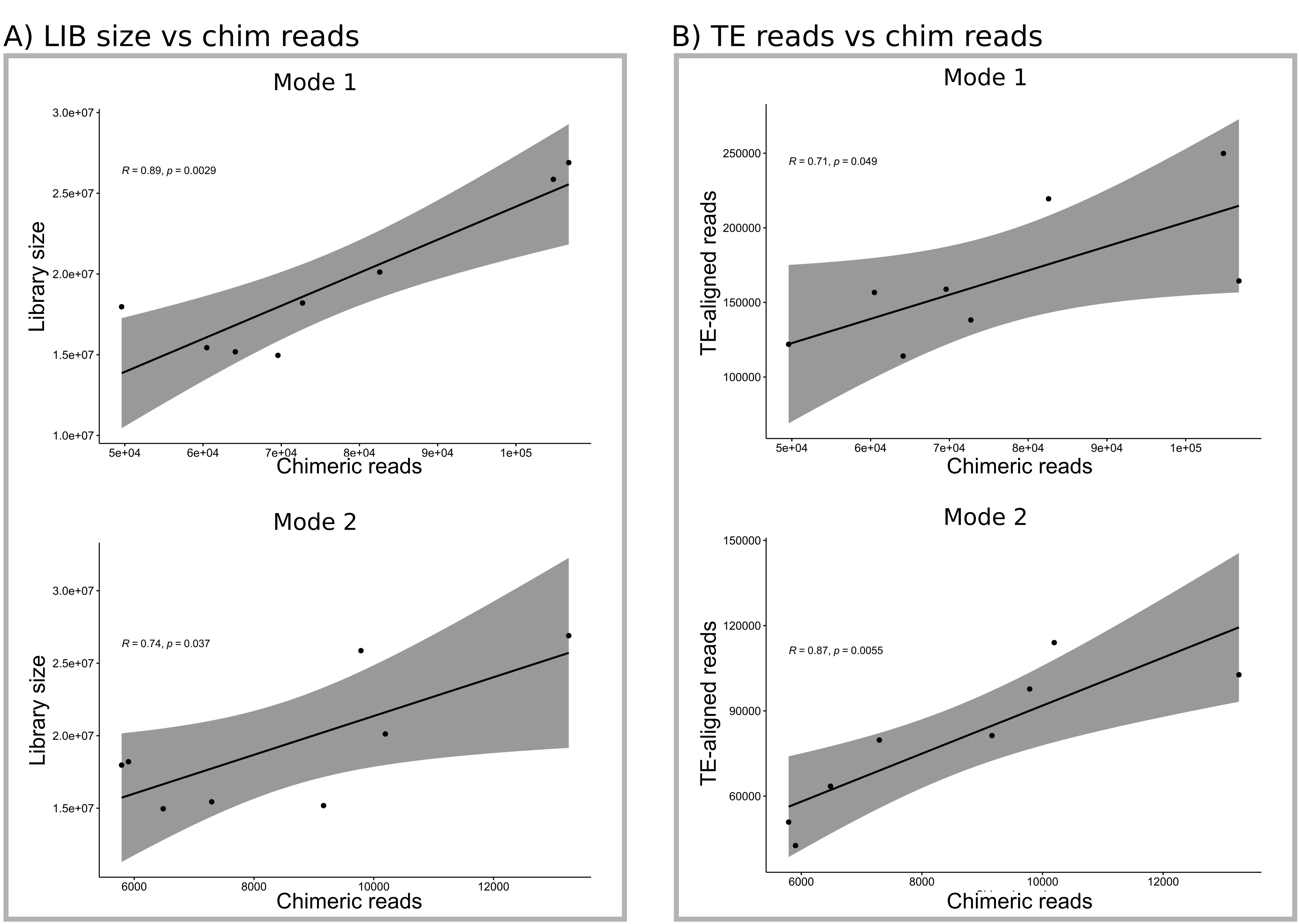


**Figure S2.3**: Positive Person correlations between: A) RNA-seq libraries size and the total of chimeric reads detected in Mode 1 (top) and Mode 2 (bottom); B) TE-aligned reads and the total of chimeric reads detected in in Mode 1 (top) and Mode 2 (bottom).

To quantify ChimeraTE replicability between RNA-seq samples in both modes, as well as the impact of changes in the coverage thresholds, we performed a comparative analysis of chimeric transcripts using two thresholds of chimeric reads, 2 and 10. Overall, Mode 1 finds 291 and 150 chimeric transcripts per strain in both replicates (Figure 2.1B), using thresholds 2 and 10 respectively, whereas Mode 2 obtains 233 and 155. These results show that by increasing the chimeric read thresholds from 2 to 10, there is a decrease of 48.45% and 33.48% in the number of detected chimeric transcripts in Mode 1 and Mode 2. Therefore, even when found in both replicates, a substantial amount of chimeric transcripts is detected with low chimeric read coverage by both modes.

**Note 3: Artifactual chimeric reads are largely reduced with RNA-seq replicability**

In both Mode 1 and Mode 2, only chimeric transcripts found in both RNA-seq replicates were considered true chimeras. Chimeric transcripts found in only one replicate may exist due to pervasive transcription in one of the replicates, the lack of read coverage in one of the replicates to predict it, or they could be artifacts of cDNA library preparation, such as template switching and hybridization of templates followed by chimeric elongation (72-74). In addition, although comprising low rates with Illumina’s traditional bridge amplification, artifactual chimeric reads might be generated due to index hopping in multiplexed sequencing (37,75). To quantify cases that may be artifacts, we aligned the RNA-seq from the four wild-type strains against their masked genomes to identify paired-end reads where each mate maps to genes from different chromosomes, or were aligned with an insert size > 500kb, and therefore are artifacts. It is important to highlight that these alignments can be inflated because they do not differentiate artifactual reads produced during sequencing, from biological fusion gene products derived from the breakage and re-joining from different chromosomes, chromosomal rearrangements (76-77) and also to reads mapping to different paralog genes or genes from the same gene family sharing high similarity. In replicate 1 and replicate 2, we found ~50,522 and ~42,303 chimeric reads representing fusions of genes that are in different chromosomes, and ~7,509, ~5,334 farther away in the genome, with at least one chimeric read (Sup. Table S3.1) Such fusions were composed in average by ~5,966 genes from different chromosomes and ~3,234 genes farther away. However, we found only 498 (8.35%) of the artifactual chimeras from different chromosomes, and 212 (6.56%) genes farther away in both RNA-seq replicates with >= 2 chimeric reads (Table S3.1). In addition, these fusion genes generated by artifacts have high expression level (average FPKM = 722 per strain), reinforcing the hypothesis that high expressed genes are more likely to produce artifacts (72).

Therefore, artifactual chimeric reads present in more than one replicate, and derived from genes and TEs, might have even lower prevalence, since the proportion of RNA-seq reads derived from TEs is very low (Figure S2.1A) in comparison to genes. Thus, in order to significantly reduce false positives, it is strongly recommended to use RNA-seq replicates, since artifactual chimeric reads exist in high frequency in highly expressed genes but only in one RNA-seq replicate. On the other hand, the proportion of the same chimeras found in more than one RNA-seq replicate with >= 2 chimeric reads is low (212 chimeras).

|  | **Replicate 1** | | | | |  | **Replicate 2** | | | | |  | **Both replicates** | | | | |
| --- | --- | --- | --- | --- | --- | --- | --- | --- | --- | --- | --- | --- | --- | --- | --- | --- | --- |
|  | ***Diff. chr.*** | |  | ***> 500kb*** | |  | ***Diff. chr.*** | |  | ***> 500kb*** | |  | ***Diff. chr.*** | |  | ***> 500kb*** | |
|  | *Reads* | *Genes* |  | *Reads* | *Genes* |  | *Reads* | *Genes* |  | *Reads* | *Genes* |  | *Reads* | *Genes* |  | *Reads* | *Genes* |
| **dmgoth101** | 99714 | 8009 |  | 18718 | 6155 |  | 19495 | 4430 |  | 1719 | 1523 |  | 6308 | 665 |  | 1422 | 256 |
| **dmgoth63** | 48539 | 6057 |  | 4361 | 2894 |  | 33556 | 5856 |  | 3558 | 2744 |  | 7301 | 314 |  | 1169 | 169 |
| **dmsj23** | 40764 | 6613 |  | 5844 | 3601 |  | 31227 | 6038 |  | 4534 | 2990 |  | 5151 | 489 |  | 1865 | 294 |
| **dmsj7** | 13072 | 3112 |  | 1113 | 1022 |  | 84934 | 7621 |  | 11525 | 4943 |  | 6950 | 527 |  | 842 | 129 |
| ***Average*** | 50522 | 5947 |  | 7509 | 3418 |  | 42303 | 5986 |  | 5334 | 3050 |  | 6427 | 498 |  | 1324 | 212 |

**Table S3.1**: Amount of artifactual chimeric reads (mates mapped in different chromosomes; or farther away > 500kb) in the two replicates, and presence of the same artifacts in both RNA-seq replicates. Green cells represent the average of genes found in both replicates with >= 2 chimeric reads.
